# Endonuclease increases efficiency of osteoblast isolation from murine calvariae

**DOI:** 10.1038/s41598-021-87716-8

**Published:** 2021-04-19

**Authors:** Yosuke Asano, Yoshinori Matsumoto, Jose La Rose, Fang He, Takayuki Katsuyama, Wang Ziyi, Shigetomo Tsuji, Hiroshi Kamioka, Robert Rottapel, Jun Wada

**Affiliations:** 1 Department of Nephrology, Rheumatology, Endocrinology and Metabolism, Okayama University Graduate School of Medicine, Dentistry and Pharmaceutical Sciences, 2-5-1 Shikata-cho, Kita-ku, Okayama, 700-8558 Japan; 2 Princess Margaret Cancer Center, University Health Network, University of Toronto, Toronto, ON Canada; 3 Department of Orthodontics, Okayama University Graduate School of Medicine, Dentistry, and Pharmaceutical Sciences, Okayama, Japan

**Keywords:** Biological techniques, Materials science

## Abstract

Bone is a highly dynamic organ that undergoes remodeling equally regulated by osteoblast-mediated bone formation and osteoclast-mediated bone resorption. To clarify the regulation of osteoblastogenesis, primary murine osteoblasts are required for an in vitro study. Primary osteoblasts are isolated from neonatal calvariae through digestion with collagenase. However, the number of cells collected from one pup is not sufficient for further in vitro experiments, leading to an increase in the use of euthanized pups. We hypothesized that the viscosity of digested calvariae and digestion solution supplemented with collagenase results in cell clumping and reduction of isolated cells from bones. We simply added Benzonase, a genetically engineered endonuclease that shears all forms of DNAs/RNAs, in order to reduce nucleic acid-mediated viscosity. We found that addition of Benzonase increased the number of collected osteoblasts by three fold compared to that without Benzonase through reduction of viscosity. Additionally, Benzonase has no effect on cellular identity and function. The new osteoblast isolation protocol with Benzonase minimizes the number of neonatal pups required for an in vitro study and expands the concept that isolation of other populations of cells including osteocytes that are difficult to be purified could be modified by Benzonase.

## Introduction

Bone is a highly dynamic organ that undergoes remodeling equally regulated by osteoblast-mediated bone formation and osteoclast-mediated bone resorption. However, the molecular and cellular mechanisms of bone dynamics have yet to be elucidated. To clarify the regulation of osteoblastogenesis, primary murine osteoblasts are required for an in vitro study. In previous studies, cells isolated from neonatal calvariae through digestion with collagenase were identified as primary osteoblasts that have the potential for differentiation and calcification^1,2^, and a protocol for osteoblast isolation has been established^3^. Briefly, neonatal calvariae are incubated in a digestion solution supplemented with collagenase and trypsin–EDTA at 37 °C and the supernatant containing digested calvarial cells is transferred to a tube. This procedure is repeated four times to obtain four different populations. The isolated calvarial cells in populations 3 and 4 including alkaline phosphatase (ALP)-expressing osteoblasts are then plated, expanded for 4–5 days, and finally collected by trypsinization for further experiments. However, the number of cells collected from one pup is not sufficient for in vitro experiments (6–10 × 10^6^ osteoblasts/20–30 pups)^3^, and the number of euthanized pups must therefore be increased.

Benzonase is a genetically engineered endonuclease from Serratia marcescens that is characterized by a dimer of 30-kDa subunits with two essential disulphide bonds^4–9^. Nucleic acids are viscous due to their high molecular weight^10^, while Benzonase shears all forms of DNAs/RNAs and reduces nucleic acid-mediated viscosity of the solution.

We hypothesized that the viscosity of digested calvariae and digestion solution supplemented with collagenase results in cell clumping and reduction of isolated cells from bones. To confirm this idea, we simply added Benzonase to the digestion solution to reduce the viscosity and investigated the collected osteoblasts. We found that nucleic acid-mediated viscosity caused by collagenase digestion reduces the number of collected osteoblasts and that addition of Benzonase increases the number of cells collected by three fold compared to that without Benzonase. Additionally, the differentiation and proliferation potential of the cells obtained with Benzonase is normal compared to that without Benzonase, indicating that Benzonase has no effect on cellular identity and function. Lastly, we histologically confirmed that Benzonase reduces viscosity of the digestion solution through shearing nucleic acids, leading to reduction of cell clumping and subsequent increase in the number of collected cells. Our results showing that the new isolation protocol with Benzonase efficiently increases the number of collected osteoblasts provides new insights into the role of endonucleases for bone research and minimizes the number of euthanized murine neonates.

## Results

### Benzonase increased the number of collected osteoblasts from calvariae

**Figure 1:**
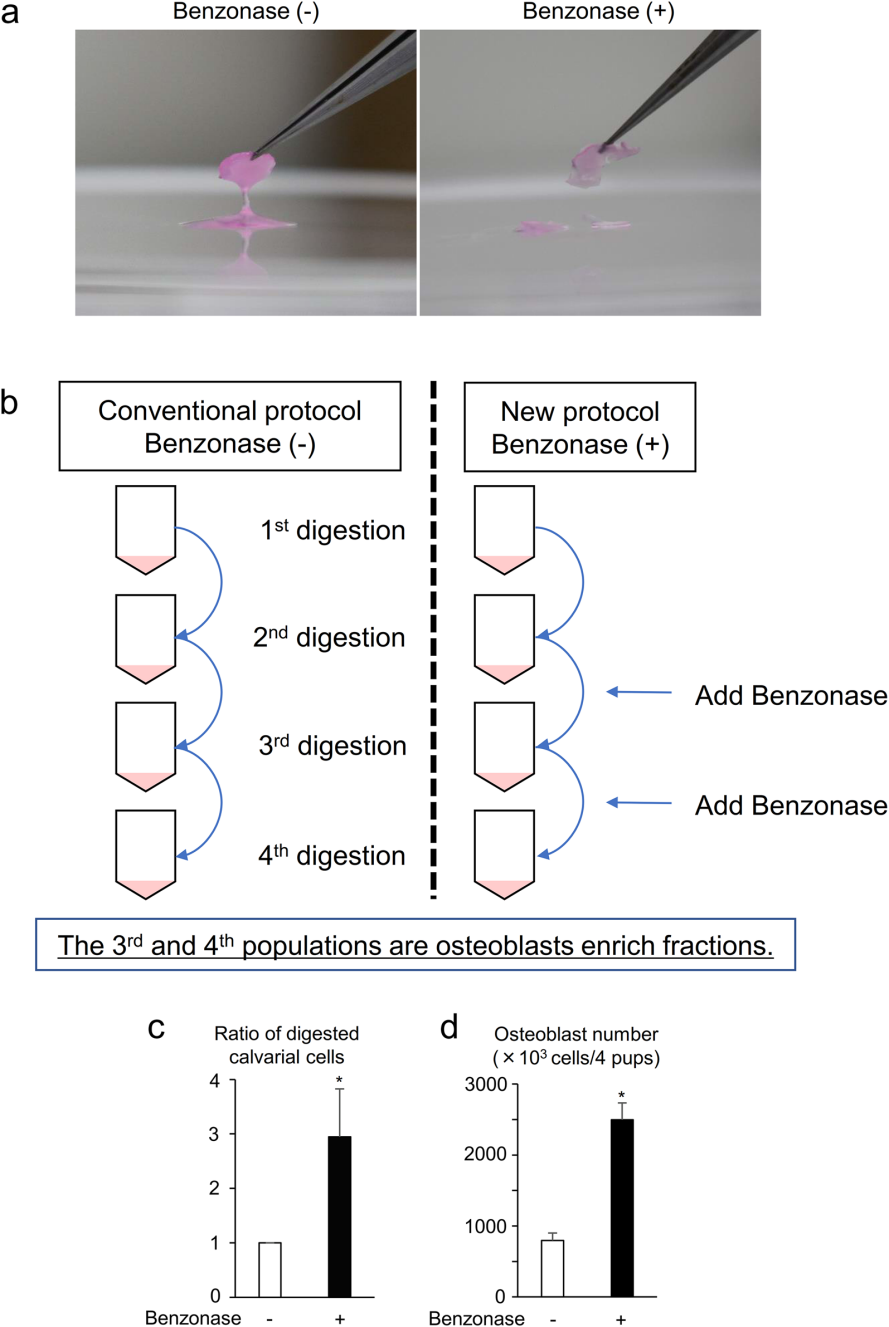
Benzonase increased the number of collected osteoblasts from calvariae. (**a**) Representative images of digested calvariae with digestion solution in the presence (right) or absence (left) of Benzonase. (**b**) Schematic process of calvarial digestion with digestion solution in the presence (right) or absence (left) of Benzonase. (**c**) The ratio of digested calvarial cells in the 3rd and 4th populations from newborn pups in the presence or absence of Benzonase. n = 5. (**d**) Total number of expanded and collected osteoblasts from calvariae from 4 newborn pups with digestion solution in the presence or absence of Benzonase. n = 3. *P* values were determined by the unpaired t-test. Data are presented as means ± SEM. *P < 0.05.

Digested calvariae in digestion solution with collagenase were viscous (Fig. 1a). We therefore first queried whether the viscosity and cell clumping were caused by nucleic acids coming from the destroyed cells that resulted in reduction of the number of collected cells. To confirm this idea, we added Benzonase to the digestion solution as described in the methods section (Fig. 1b) and observed the disappearance of viscosity and a threefold increase in the ratio of digested calvarial cells and the number of collected osteoblasts compared to those without Benzonase (Fig. 1a,c,d). These results suggest that Benzonase in the digestion solution reduces viscosity of digested bones, leading to an increase in the collection of calvarial osteoblasts.

### Benzonase did not affect the diversity of digested calvarial cells

**Figure 2:**
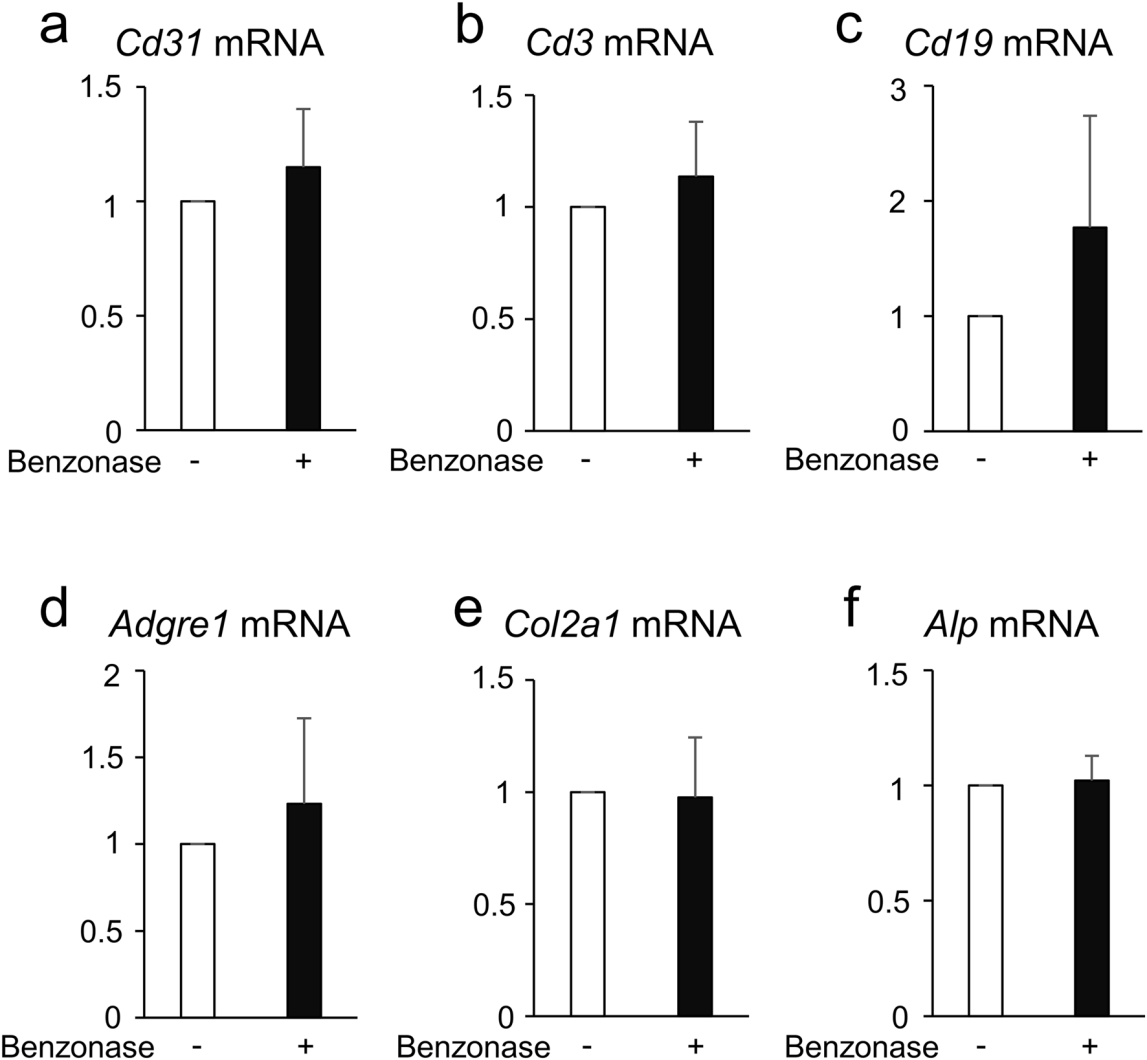
Benzonase did not affect the diversity of digested calvarial cells. (**a**–**f**) qPCR analysis of *Cd31* (**a**), *Cd3* (**b**), *Cd19* (**c**), *Adgre1* (**d**), *Col2a1* (**e**) and *Alp* (**f**) mRNA expression in digested calvarial cells shown in Fig. 1c. n = 3. *P* values were determined by the unpaired t-test. Data are presented as means ± SEM. *P < 0.05.

We next queried whether addition of Benzonase affected the diversity of isolated calvarial cells during digestion. To address this question, we investigated the mRNA expression levels of *Cd31* (endothelial cells), *Cd3* (T lymphocytes), *Cd19* (B lymphocytes), *Adgre1* (macrophages), *Col2a1* (chondrocytes) and *Alp* (osteoblasts) in digested calvarial cells and observed that addition of Benzonase did not affect the expression of these transcripts (Fig. 2a–f). These results indicate that addition of Benzonase increases the total number of digested calvarial cells including osteoblasts without affecting cellular diversity.

### Benzonase did not affect osteoblast differentiation and proliferation

**Figure 3:**
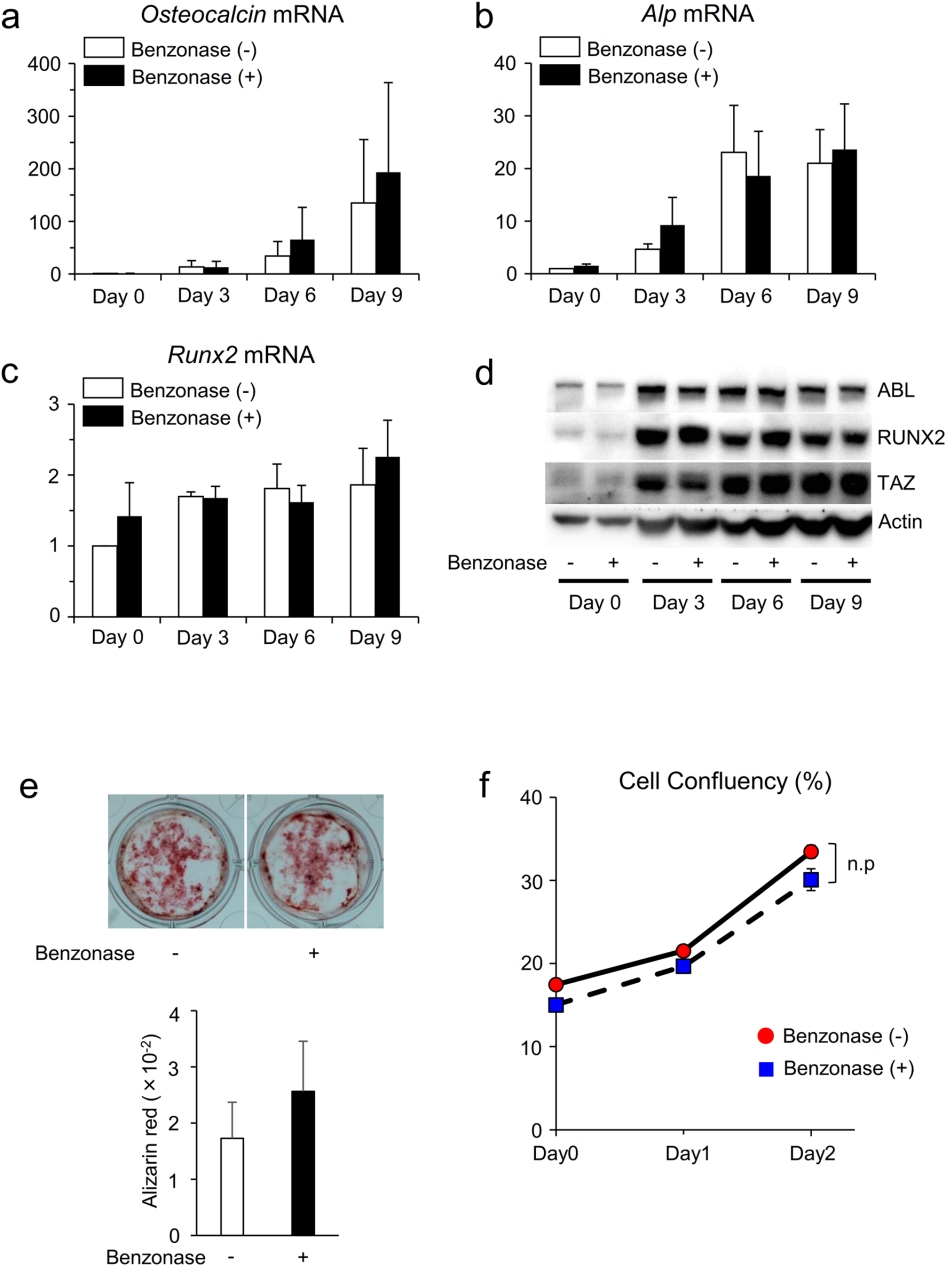
Benzonase did not affect osteoblast differentiation and proliferation. (**a**–**c**) qPCR analysis of *Osteocalcin* (**a**), *Alp* (**b**) and *Runx2* (**c**) mRNA expression in primary murine osteoblasts expanded and collected in Fig. 1d and cultured in an osteogenic medium for 3–9 days. n = 3. (**d**) Whole cell lysates from cells in (**a**–**c**) were probed with the indicated antibodies for Western blot analysis. (**e**) Primary murine osteoblasts expanded and collected in Fig. 1d were cultured in an osteogenic medium for 21 days and stained with alizarin red S solution (upper). Mineralization of the cells was evaluated by the absorbance at 405 nm (lower). n = 4. (**f**) Growth curves of primary murine osteoblasts expanded and collected in Fig. 1d and cultured in a growth medium for 2 days. *P* values were determined by the unpaired t-test. Data are presented as means ± SEM. *P < 0.05.

We next queried whether Benzonase affects osteoblast differentiation and proliferation, and we found that the mRNA expression levels of *Osteocalcin*, *Alp* and *Runx2* in collected osteoblasts with Benzonase were similar to those in cells without Benzonase during osteoblastogenesis (Fig. 3a–c). We have reported that RUNX-TAZ complex formation promoted by the non-receptor tyrosine kinase ABL is critical for osteoblast differentiation and proliferation^11^. We therefore investigated the protein expression levels of ABL, TAZ and RUNX2 and the potential of mineralization and proliferation in primary murine osteoblasts, and we found no difference in the presence or absence of Benzonase (Fig. 3d–f). These results indicate that addition of Benzonase during calvarial digestion does not affect osteoblast function and proliferation.

### Benzonase shears nucleic acids and separates osteoblasts from digested viscous bones

**Figure 4:**
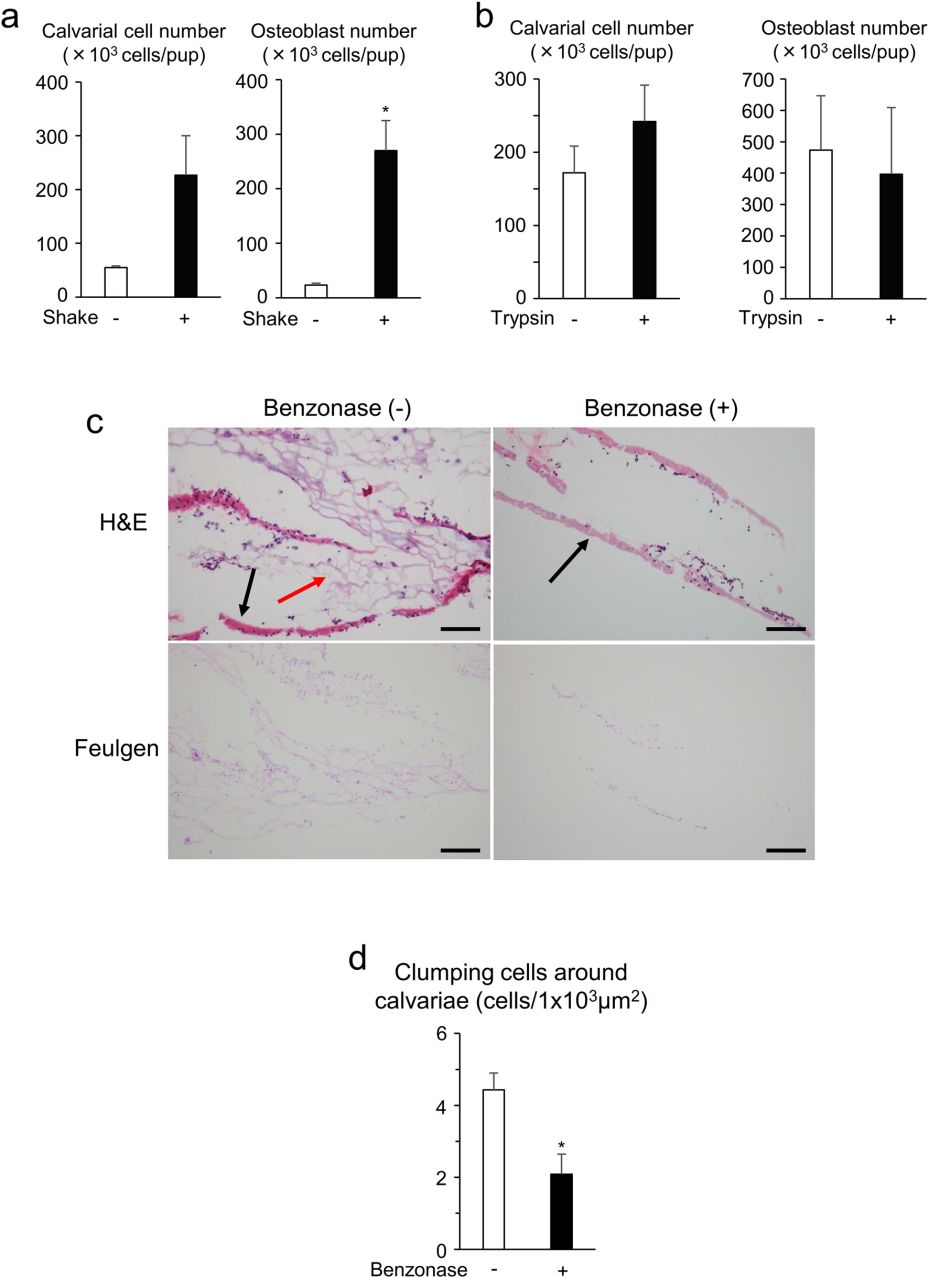
Benzonase shears nucleic acids and separates osteoblasts from digested viscous bones. (**a**, **b**) Neonatal calvariae were digested in the digestion solution supplemented with collagenase in the presence or absence of mechanical agitation in the shaking water bath (**a**) or trypsin (**b**). Digested calvarial cells were plated, expanded for 4–5 days, and collected as osteoblasts by trypsinization. Calvarial cells and osteoblasts from one pup’s calvaria were counted. (**c**) H&E (upper) and DNA Feulgen (lower) staining of newborn pups’ calvariae digested in the presence or absence of Benzonase. Black and red arrows indicate calvarial bones and connective tissues, respectively. Scale bars: 100 μm. (**d**) Histomorphometric analysis of osteoblast number per bone surface of calvariae in (**c**). n = 3. *P* values were determined by the unpaired t-test. Data are presented as means ± SEM. *P < 0.05.

To clarify the mechanism by which digested calvariae were viscous, we investigated the requirement of mechanical agitation and trypsin during digestion. We observed that digestion with mechanical agitation increased the viscosity of calvarial bones and the number of digested calvarial cells and collected osteoblasts after expansion compared to those without shaking (Fig. 4a). On the other hand, addition of trypsin did not affect the number of digested calvarial cells and collected osteoblasts (Fig. 4b). These results suggest that mechanical agitation by shaking, but not trypsin, is required for optimum osteoblast isolation with collagenase and that addition of Benzonase reduces the viscosity of digested bones, leading to an increase in collection of osteoblasts.

Lastly, to confirm our observations, we performed histological analysis. H&E staining of digested calvariae from newborn pups revealed that isolated calvarial cells were aggregated around the surfaces of the bones, while Benzonase treatment cleared these cells (Fig. 4c). Of note, DNA Feulgen staining of calvariae showed that nucleic acids with aggregated cells were sheared and cleared by Benzonase (Fig. 4c,d). These results conclusively demonstrate that Benzonase in the digestion solution shears nucleic acids during digestion, resulting in reduction of cell clumping and enhancement of osteoblast isolation.

## Discussion

In the present study, we showed that Benzonase in combination with collagenase and mechanical agitation increases the number of osteoblasts collected from digested newborn calvariae. Benzonase shears DNA/RNA, which causes cell clumping around digested bones, and separates the cells, leading to an increase in the number of isolated calvarial cells. Additionally, Benzonase treatment does not affect osteoblast differentiation, mineralization or proliferation.

Since the molecular mechanisms of osteoblast differentiation and proliferation remain unclear, primary osteoblast isolation from neonatal calvariae is required to perform an in vitro study. Methodology for osteoblast isolation has been established, and at least 20–30 newborn pups are decapitated to collect calvariae for subsequent digestion. However, we observed cell clumping around digested calvariae, leading to reduction in the efficiency of calvarial cell isolation. Histological analysis with DNA Feulgen staining revealed that DNA/RNA from digested calvariae causes viscosity and cell clumping, which was cleared by Benzonase. Benzonase requires divalent magnesium cations for its endonuclease activity and it is used for separation of peripheral blood mononuclear cells (PBMCs)^12^. That previous study prompted us to use Benzonase during osteoblast isolation through calvarial digestion. Our results showing that Benzonase increases collected osteoblasts could expand the concept that Benzonase is able to minimize the number of neonatal pups required for an in vitro study and that isolation of other populations of cells including osteocytes that are difficult to be purified could be modified by Benzonase.

## Materials and methods

### Osteoblast isolation and cultures

All animal studies were approved by the Animal Research Council at Okayama University, Okayama, Japan. Animal experiments were carried out in compliance with the ARRIVE guidelines (http://www.nc3rs.org.uk/page.asp?id=1357) and the NIH guidelines (Guide for the Care and Use of Laboratory Animals). Neonatal calvariae-derived osteoblasts from C57BL/6 J mice (CLEA Japan Inc., Osaka, Japan) were harvested and cultured as described previously (Fig. 1b)^3^. Briefly, neonatal murine pups were euthanized by decapitation, and the heads were placed in a petri dish with PBS. The skin of each head was cut away, and the calvariae were cut and washed with PBS in a petri dish. The calvariae were incubated in 4 ml of digestion solution supplemented with 2.56 mg of collagenase II (250–255 units/mg, Thermo Fisher Scientific 17101015) and 0.8 ml of trypsin–EDTA (0.25%, Sigma T4049) at 37 °C in a shaking water bath. During the incubation, the calvariae were shaken by hand for a few seconds. After incubation for 20 min, 700 μl FBS was added to the cell suspension to inhibit trypsin activity. The calvariae were washed with 3 ml DMEM without FBS and shaken well and then the supernatant was transferred to a tube containing the cell suspension. This cell population was termed population number 1. The calvariae were transferred to a new digestion solution to repeat the previous steps in order to obtain population number 2. The entire procedure was repeated four times to obtain populations 1–4. The isolated calvarial cells in populations 3 and 4 including alkaline phosphatase (ALP)-expressing osteoblasts were then plated, expanded for 4–5 days, and finally collected by trypsinization for further experiments. In this study, 2 μL of Benzonase (≧ 250 units/µl, Sigma E1014) was added to 4 ml of digestion solution before adding FBS in the 3rd or 4th session of calvarial digestion, and collected osteoblasts with or without Benzonase were analyzed. Osteoblast differentiation was induced by culturing cells in an osteogenic medium (α-MEM containing 10% FBS, 100 μg/ml ascorbic acid and 10 mM β-glycerophosphate) for 21 days as described previously^11,13^. Alizarin red staining of mineralization was accomplished by cell fixation in 4% formaldehyde for 30 min followed by staining with 0.1% Alizarin Red-S solution (pH 4.8) for 20 min. Alizarin red dye was extracted with 5% formic acid, and the absorbance at 405 nm was determined with a microplate reader as described previously^14^.

### Histomorphometry

The in vivo isolated cells were analyzed from sections of neonatal calvariae that had been digested with digestion solution in the presence or absence of Benzonase, embedded in optimal cutting temperature compound, and stained with H&E or DNA Feulgen staining, which specifically stains deoxyribonucleoprotein. We examined the number of cells and the amount of viscous substance surrounding bones by H&E staining. The number of cells was automatically estimated by counting the hematoxylin-stained nuclei using “Analyze Particles” function in ImageJ/Fiji and normalized by the reference area. Before analysis of the substance area and cell number, the images were pre-processed by white balance correction and "subtract background”. Then they were cropped according to ROI selection.

### Reagents and antibodies

Unless stated otherwise, all chemicals were purchased from Sigma. Antibodies were obtained from the following sources: anti-ABL, anti-TAZ (BD Pharmingen), anti-RUNX2 (MBL International) and anti-Actin (Santa Cruz Biotechnologies). Halt™ Protease and Phosphatase Inhibitor Cocktail was from Thermo Fisher Scientific.

### RNA extraction and quantitative real-time PCR analysis

Total cellular RNA was extracted using an RNeasy Plus Mini Kit (QIAGEN). A High Capacity cDNA Reverse Transcription Kit (Thermo Fisher Scientific) was used for reverse transcription, and qPCR was performed on a Step One Plus Real-Time PCR System (Applied Biosystems) according to the manufacturer’s protocol. The sequences of primers are as follows: mouse *Rpl19* (forward primer, 5′-CTG AAG GTC AAA GGG AAT GTG-3′; reverse primer, 5′-GGA CAG AGT CTT GAT GAT CTC-3′), mouse *Alp* (forward primer, 5′-GCT GAT CAT TCC CAC GTT TTC-3′; reverse primer, 5′-CTG GGC CTG GTA GTT GTT GT-3′), mouse *Osteocalcin* (forward primer, 5′-CTG ACA AAG CCT TCA TGT CCA A-3′; reverse primer, 5′-GCG CCG GAG TCT GTT CAC TA-3′), mouse *Runx2* (forward primer, 5′-GCT ATT AAA GTG ACA GTG GAC G-3′; reverse primer, 5′-CAC GTC AGT GAT GGC AGG TAG C-3′), mouse *Cd3* (forward primer, 5′-ATG CGG TGG AAC ACT TTC TGG-3′; reverse primer, 5′-GCA CGT CAA CTC TAC ACT GGT-3′), mouse *Cd19* (forward primer, 5′-GGA GGC AAT GTT GTG CTG C-3′; reverse primer, 5′-ACA ATC ACT AGC AAG ATG CCC-3′), mouse *Adgre1* (forward primer, 5′-GAA GCA TCC GAG ACA CAC AC-3′; reverse primer, 5′-TTG TGG TTC TGA ACA GCA CG-3′), mouse *Cd31* (forward primer, 5′-GAG CCC AAT CAC GTT TCA GTT-3′; reverse primer, 5′-TCC TTC CTG CTT CTT GCT AGC-3′) and mouse *Col2a1* (forward primer, 5′-CAC ACT GGT AAG TGG GGC AAG A-3′; reverse primer, 5′-GGA TTG TGT TGT TTC AGG GTT CG-3′). The relative expression of each mRNA was calculated by the ΔCt method.

### Western blot analysis

Cells were lysed with RIPA buffer (50 mM Tris [pH 7.5], 150 mM NaCl, 1% NP40, 0.1% SDS, 0.25% sodium deoxycholate, 1 mM EDTA) supplemented with protease and phosphatase inhibitors. Lysates were cleared by centrifugation at 4 °C for 10 min at 14,000 rpm. For Western blotting, protein in whole cell lysates was resolved by SDS-PAGE and transferred to a PVDF membrane (Immobilon; Millipore). Membranes were blocked in 5% BSA or 5% nonfat dried milk in PBST (PBS + 0.1% Tween-20) as described previously^15^. Images shown are representative of three independent experiments.

### Cell growth assays

Primary murine osteoblasts were plated in a regular growth medium and placed into the INCUCYTE™ Kinetic Imaging System (Essen Bioscience) to monitor cell growth and percent cell confluence.

### Statistics

All results are shown as means ± SEM of data from at least three separate experiments. The data were subjected to the unpaired t-test with JMP® 7 (SAS Institute Inc, USA) to determine differences. *P* values < 0.05 were accepted as statistically significant.

## Supplementary Information

Supplementary Information
