# Supplementary figures and images for: Endonuclease increases efficiency of osteoblast isolation from murine calvariae
Source: Sci Rep. 2021 Apr 19;11:8502. doi: 10.1038/s41598-021-87716-8 (PMC8055883; doi:10.1038/s41598-021-87716-8)

# Fig. 3d

Original gels of fig.2b.

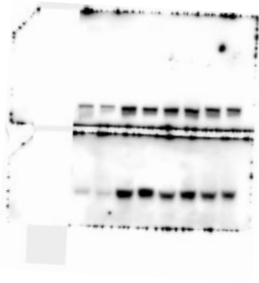

ABL

RUNX2

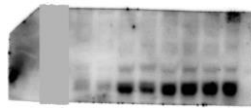

TAZ

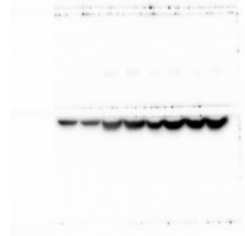

Actin

Supplement: Supplementary file 1 — Supplementary Information [file 41598_2021_87716_MOESM1_ESM.pdf]
